# Initial exploration of the discriminatory ability of the PetPace collar to detect differences in activity and physiological variables between healthy and osteoarthritic dogs

**DOI:** 10.3389/fpain.2022.949877

**Published:** 2022-09-06

**Authors:** Avery Rowlison de Ortiz, Beatriz Belda, Jon Hash, Masataka Enomoto, James Robertson, B. Duncan X. Lascelles

**Affiliations:** ^1^Translational Research in Pain Program, Department of Clinical Sciences, College of Veterinary Medicine, North Carolina State University, Raleigh, NC, United States; ^2^Office of Research, College of Veterinary Medicine, North Carolina State University, Raleigh, NC, United States; ^3^Comparative Pain Research and Education Center, College of Veterinary Medicine, North Carolina State University, Raleigh, NC, United States; ^4^Thurston Arthritis Center, University of North Carolina (UNC) School of Medicine, Chapel Hill, NC, United States; ^5^Department of Anesthesiology, Center for Translational Pain Research, Duke University, Durham, NC, United States

**Keywords:** canine, physical activity monitor, osteoarthritis, mobility, pain, accelerometry

## Abstract

**Background:**

Accelerometry has been used to evaluate activity in dogs with osteoarthritis (OA) pain, especially in relation to effect of treatment; however no studies have compared accelerometry-measured activity in dogs with OA-pain and healthy dogs. The aims of this study were to (1) compare activity output from the PetPace collar with the validated Actical monitor and (2) determine if PetPace collar outputs (overall activity, activity levels, body position, and vital signs) differed between healthy dogs and dogs with OA-pain.

**Methods:**

This was an observational, non-interventional study in healthy dogs and dogs with OA-pain. All dogs were outfitted with the PetPace collar and the Actical monitor simultaneously for 14 days. Output from these devices was compared (correlations), and output from the PetPace device was used to explore differences between groups across the activity and vital sign outputs (including calculated heart rate variability indices).

**Results:**

There was moderate correlation between the PetPace collar and Actical monitor output (*R*^2^ = 0.56, *p* < 0.001). Using data generated by the PetPace collar, OA-pain dogs had lower overall activity counts and spent less time standing than healthy dogs. Healthy dogs spent more time at higher activity levels than OA-pain dogs. Certain heart rate variability indices in OA-pain dogs were lower than in healthy dogs.

**Conclusions and clinical relevance:**

The results of this study suggest that the PetPace collar can detect differences between healthy dogs and those with OA-pain, and that OA-pain negatively impacts overall activity levels in dogs, and especially higher intensity activity.

## Introduction

Osteoarthritis (OA) in dogs is a progressive, degenerative joint disease which can result in chronic pain (1). The prevalence of OA among dogs is poorly understood. One survey from 1997 estimated that the condition affects 20% of dogs over the age of 1 year in North America (2). A recent study of dogs presenting to general practices suggested the prevalence of OA and associated clinical signs may be as high as 37% (3). Though strong data regarding prevalence of this disease are lacking, the data do suggest that OA is likely a common disease among dogs.

The disease of OA can be associated with pain (as determined by various means), and this has been referred to as “OA-associated pain,” or “OA-pain” (4). Mainstays of treatment of OA-associated pain (OA-pain) include the use of non-steroidal anti-inflammatory drugs (NSAIDs), other analgesics, supplements, and weight management (5). Despite how common OA-pain is in dogs, data surrounding efficacy of many suggested treatments are rather limited, probably partly due to the difficulty in measuring the impact of OA-pain. Assessing response to therapy has historically relied upon the veterinarian's assessment at recheck visits, however subjective lameness evaluations have been shown to have low concordance with more objective measures such as force plate analysis (6). Kinetic gait analysis has been used as an objective outcome measure to diagnose lameness and assess response to therapy, but equipment availability is limited, making routine implementation difficult (7, 8). More recently, clinical metrology instruments (CMIs, or client reported outcome measures) including the Canine Brief Pain Inventory (CBPI), Liverpool Osteoarthritis in Dogs (LOAD), and Sleep and Nighttime Restlessness Evaluation (SNoRE) questionnaires have been utilized in assessing response to treatment (9–11). These proxy assessments performed by owners are subjective and can be affected by various biases (12).

Accelerometry using physical activity monitors offers an opportunity for objective and non-invasive measurement of activity in companion animals. With the general acceptance that OA-pain negatively affects activity, accelerometry has been investigated for use in companion animals as an outcome measure of the efficacy of treatments for OA-pain (13–17). Most work has been performed with the Actical physical activity monitor which has been validated as a measure of activity and distance moved in dogs (18) and has been used to evaluate response to treatment (10, 13, 19). Though several studies have used accelerometry to evaluate healthy dogs or dogs with OA, and response to treatment, there are no studies directly comparing physical activity measurements in clinically healthy dogs to those of dogs with OA.

There are now several commercially available physical activity monitors for dogs. One such physical activity monitor is the PetPace collar which includes an accelerometer and incorporates technology that allows for certain vital signs to be recorded. Data are recorded by the collar and uploaded to a cloud-based server over WiFi. There is a dashboard with summary statistics that both owners and veterinarians can access online. The validity of the output from the PetPace collar and the summary statistics have not been made public or been published. In a recent pilot study, physical activity count output from the PetPace collar was found to have moderate correlation with the Actical monitor output in healthy dogs (20), suggesting that the physical activity data output from the PetPace collar are a valid measure of activity of the dog.

The aims of this study were to (1) compare activity counts from the PetPace collar to the validated Actical monitor in a larger data set than previously described (20); and (2) determine whether the PetPace collar activity, activity levels, body position, and vital sign measurements differ between healthy dogs and dogs with OA-pain. We hypothesized that the PetPace collar output would correlate highly (*R*^2^ ≥ 0.70) with the Actical monitor output and that differences in activity metrics between healthy dogs and dogs with OA-pain would be detected by the PetPace collar.

## Materials and methods

### Study design

This was an observational, non-interventional study using physical activity monitoring devices in healthy dogs and dogs with OA-pain. All dogs were outfitted with two devices—a PetPace collar (PetPace, LLC, Burlington, MA) and an Actical monitor (Philips Respironics, Bend, OR, USA) that was mounted onto the PetPace collar for 14 days. The dogs' owners were instructed to maintain a daily diary during the study period, and they completed CMIs on days 0 and 14. Outcome measures consisted of activity count output from each accelerometer as well as derived indices and measured vital signs from the PetPace collar. All study procedures were approved by the Animal Care and Use Committee at North Carolina State University (IACUC #17-110-O). Written, informed consent was obtained from all owners for the participation of their dogs in this study.

Dogs were housed in the Veterinary Hospital General Housing ward at the North Carolina State University College of Veterinary Medicine (NCSU-CVM) on days 0 and 14, and they were discharged to the home environment after all procedures had been completed that day. Dogs were provided bedding, toys, and water while in the hospital.

Reporting of the study follows CONSORT guidelines (21).

### Study site and personnel

This study was conducted at the NCSU-CVM in Raleigh, NC. All study investigators were veterinarians and technical support was provided by licensed veterinary technicians.

### Study timeline

**Figure 1:**
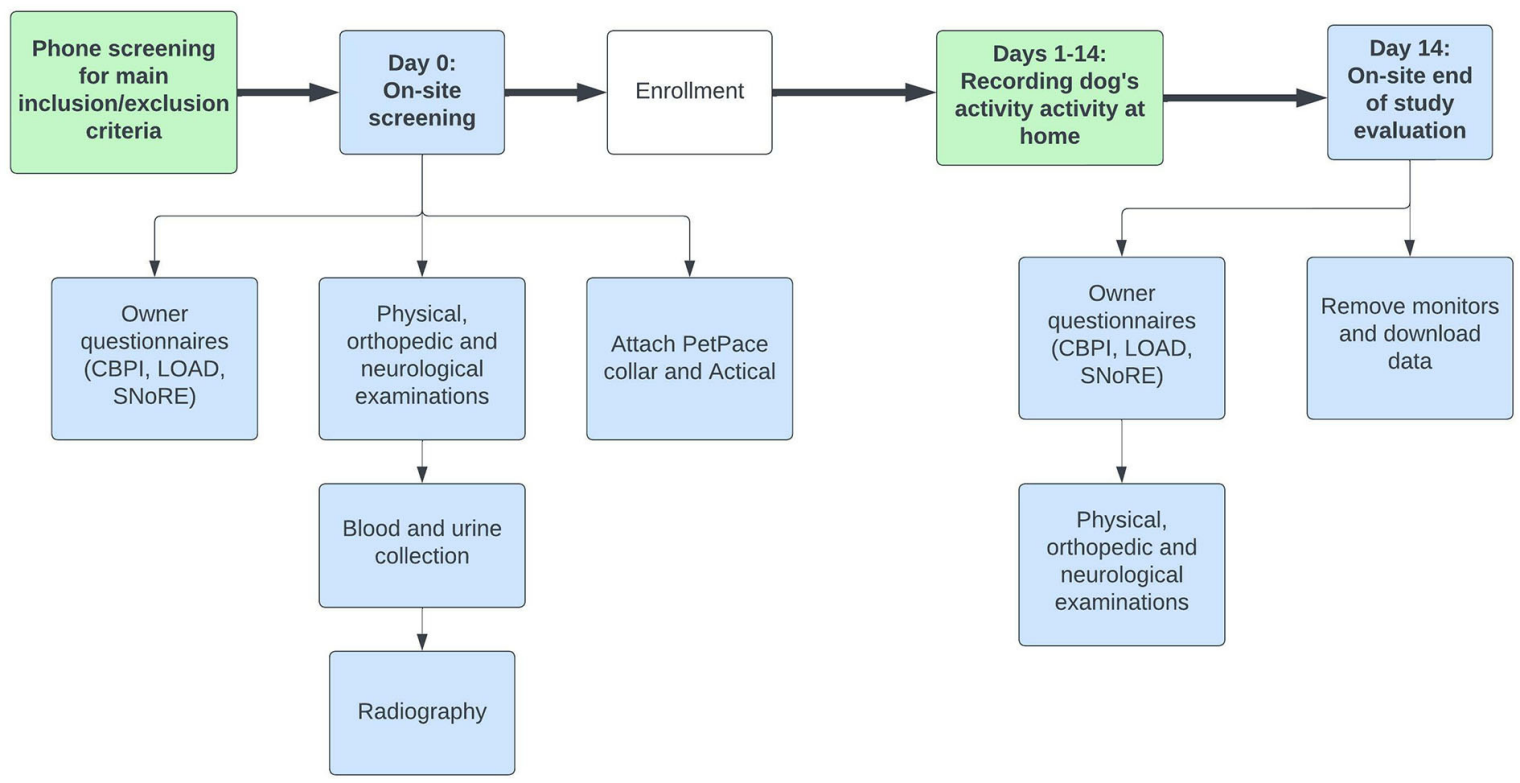
Schematic diagram showing study timeline and activities.

The study timeline is outlined in Figure 1. Dogs were pre-screened by telephone or during an in-person visit to the NCSU-CVM. The dogs were then screened (day 0, screening visit) wherein physical, orthopedic, and neurologic examinations were performed, hematology, serum chemistry and urinalysis were conducted, and radiographs of all painful joints were obtained. Dog owners completed CMIs (described below) and the PetPace collar and Actical activity monitor were attached to the dog. On day 14, dogs were returned to the NCSU-CVM for physical, orthopedic, and neurologic examinations, and owners completed the CMIs. The activity monitors were removed, and data were downloaded.

### Inclusion/exclusion criteria

Client-owned adult dogs (>1 year) of either sex, any breed, weighing between 10 and 70 kg were recruited. Dogs were recruited into two groups: dogs with owner-reported mobility impairment and OA-pain and healthy dogs (control). Dogs were pre-screened to identify those with OA-pain as well as healthy dogs with similar age and size characteristics. The goal was to create similar groups of dogs with respect to demographics, but the study was not designed as a case-matched controlled study.

Dogs eligible for enrollment in the healthy group were required to have no signs of chronic pain (e.g., joint pain, muscle atrophy). Dogs eligible for enrollment in the OA-pain group were required to have owner-perceived mobility impairment for at least 6 months, pain (based on the veterinarian's examination) in at least one joint or spinal segment, and radiographic evidence of OA in at least one appendicular joint that was painful. If OA was due to a ruptured cruciate ligament, the rupture must have occurred at least 6 months prior to the date of inclusion. Owners were not aware of details of the inclusion criteria prior to a decision being made about enrollment. The overall aim was to create two groups of dogs: one group that was generally healthy with no signs of OA-pain and another group of dogs that were generally healthy but displaying obvious signs of OA-pain.

Dogs were excluded from the study if they did not meet the inclusion criteria, had a concomitant disease that was considered to be contributing to joint pain or overall disability (e.g., joint instability, neurological disease, surgical alteration such as femoral head and neck excision, symptomatic cardiac disease, etc.), or had undergone any surgery within 3 months prior to this study. Other exclusion criteria included being pregnant, receiving corticosteroids of any type (oral, injectable or topical), NSAIDs or other drugs considered to be analgesic (e.g., amantadine, gabapentin, or tricyclic antidepressants with a 3-week wash out period required for any analgesics/putative analgesics), or nutritional supplements that had been administered for <6 weeks. Dogs with abnormal hematology, clinical chemistry, or urinalysis results that had clinical significance were also excluded from this study.

### Orthopedic evaluation

Every limb was examined, and joints were graded for pain, crepitus, effusion, and thickening. Spinal column segments were examined and graded for pain. See Supplementary File 1 for joint assessment. Scores for pain ranged from 0 to 4; these scores were used to create a Total Pain Score (sum of individual pain scores for each joint) with a range of 0–84. Assessments for crepitus, effusion, thickening, and range of motion were recorded, but not used in analysis.

### Radiographic evaluation

Radiography was used to confirm the diagnosis of OA. Only joints where a pain response (behavioral indicator of aversion to joint manipulation such as withdrawal) was detected during orthopedic evaluation were radiographed. Radiographic interpretations were performed by a board-certified veterinary radiologist unaware of the results of the orthopedic examination (they had no access to joint pain data).

### Clinical metrology instruments

Owner-completed CMIs were used as previously described (9–11). These CMIs have been shown to differentiate dogs with OA-pain from those without, and the Liverpool Osteoarthritis in Dogs (LOAD) (10, 22) and Canine Brief Pain Inventory (CBPI) (9) have been shown to be a valid measure of the impact of OA-pain in dogs. Sleep Nighttime and Restlessness Evaluation Score (SNoRE) Questionnaire version 1.0 was used to collect the data, but the data were analyzed using version 2.0 where question 6 is omitted due to improved sensitivity of the instrument (23).

The CMIs were completed by the dog owner on day 0 and day 14. They were completed by the same owner for each dog, and owners were not given access to their prior assessment. Day 0 CMIs were used to assist with selection of dogs (see above) and to explore the relationship between owner-scored impairment and activity. Day 14 CMI scores were only used to determine if there had been any perceived change in a dog's status over the 2 weeks.

### Accelerometry

On day 0, the Actical and PetPace physical activity monitors were placed on the dog and remained on the dog for 14 days. The PetPace device is a collar with integrated monitors, designed for cats and dogs. It records data related to activity, body position, and vital signs (pulse rate, respiratory rate, temperature). The PetPace collar uses a tri-axial accelerometer to collect activity data with a sampling rate of 1 Hz. Additionally, the 3D-accelerometer data are used to determine the orientation of the collar which in turn is used to determine body position. Body position is recorded when there is no activity detected for 6 s (24). The collar also records pulse and respiratory rates from pulse waves using acoustic sensors situated on the inside of the collar, facing toward the neck. Because acoustic sensors are used, vital sign data can be influenced by activity, sounds such as barking or poor collar fit (24). Data collected by the PetPace collar is synchronized wirelessly to a gateway connected to an Ethernet port, and the data are then uploaded automatically to a cloud-based server. The epoch length for PetPace is fixed by the manufacturer, and values for activity data and body position are obtained every 128–134 s. For vital signs, the epoch length selected was 15 min. It is important to point out that no validation of the output from the PetPace collar has been published or made publicly available. Therefore, this study took the manufacturers claims at face value and determined whether the output differed between healthy dogs and dogs with OA-pain.

**Figure 2:**
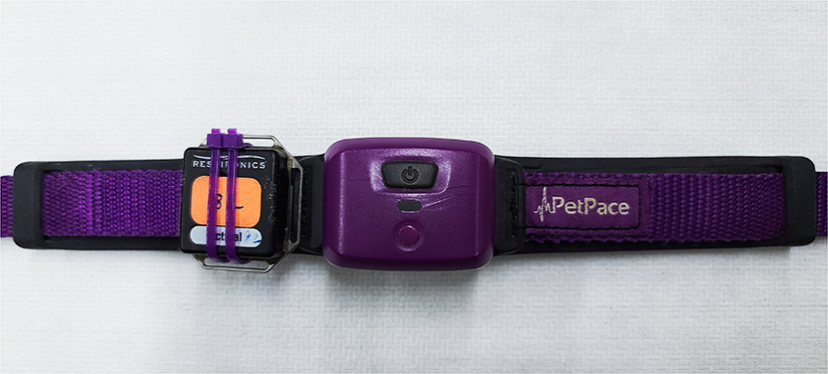
Picture depicting the configuration of the Actical monitor on the PetPace collar used in this study. The Actical monitor was fixed to the PetPace collar using zip ties, which were then covered with VetWrap to prevent injury from the sharp ends of the zip ties.

The Actical monitor was mounted on the PetPace collar using zip ties and VetWrap (Figure 2). The Actical monitor is a commercially available uniaxial accelerometer that has been shown to be a valid surrogate measure of distance moved in dogs (18). The sampling rate was set to 30 Hz, and the epoch length was set to 60 s.

There were 17 Actical monitors and 18 PetPace collars used in this study. Each PetPace collar and Actical monitor was numbered and associated with a unique serial number. The Actical monitors and PetPace collars were assigned to each patient depending on the availability of the devices and the size of the PetPace collar required for an individual dog.

### Owner diary

Owners were asked to complete a diary over the 14-day study period (Supplementary File 2). The times that the household awoke and went to bed each day were recorded in the diary; these data were used to partition the data into daytime and nighttime intervals. Owners were also asked to report any time that the collar was not attached to the dog and its circumstances.

### Collar-based outcome measures

The primary outcome measures were the PetPace and Actical accelerometry data. PetPace accelerometry data were compared to those collected by the Actical monitor, and accelerometry data from the PetPace collar were used to compare healthy and OA-pain dogs. The secondary outcome measures were the data collected by the PetPace collar regarding body position, heart rate variability, and vital signs.

#### Activity

Accelerometry output (“physical activity counts”) from each device were summed for each hour, for each dog, and used to assess the correlation between the output of the two devices. Mean activity counts per hour recorded by the PetPace collar over the 14-day period were used to compare healthy and OA-pain groups.

The dashboard output of the PetPace report shows the time that each dog spent in the different levels of activity (rest, low, medium, high) in a histogram format, however we obtained access to the raw data where the time the dog spent in each level of activity was provided. The cut points for each level of activity are considered confidential by PetPace and were not disclosed. These measurements were calculated for daytime, nighttime, weekday, and weekend periods and expressed as a single value for the entire study period. The percentage of readings that were zero were also recorded. Additionally, the average of the hourly activity counts over the study period were calculated.

#### Position

The PetPace collar purportedly detects the following positions: lying sternal, lying on the left side, lying on the right side, lying on the back, sitting, eating, standing, and undefined. Position is recorded by the device when the dog maintains this position for more than 6 s without activity (movement) being detected. In this study, all lying positions were grouped and compared with standing and sitting between groups for nighttime and daytime. The relative percentage of time spent in each position was expressed as a percentage of the total time that any position was recorded.

#### Vital signs

The PetPace collar records pulse rate and respiratory rates. The collar was set to take pulse and respiratory rates every 15 min. Pulse and respiration can only be reliably recorded if the dog is not moving as there is degradation of the signal during activity. The minimum, maximum, average, and standard deviation of the pulse and respiratory rates were calculated for daytime and nighttime periods. The percentages of pulse and respiratory rate readings relative to the maximum total number that could have been recorded during the study period were calculated and reported. Heart Rate Variability (HRV) parameters were calculated by proprietary algorithms applied to heart rate measurements collected by the collar, and derived parameters include vasovagal tone index (VVTI), the standard deviation of the average normal to normal inter-beat intervals for each 5 min segment of a 24 h HRV recording (SDANN), the mean of the SDANN (SDANN Index), the standard deviation of normal to normal inter-beat intervals (SDNN), and the integral of the density of the respiratory rate interval histogram divided by its height (triangular index). These measurements have not been validated in published studies.

### Sample size estimation and statistical analysis

No work had been performed comparing measurable activity indices in healthy dogs and dogs with OA-pain, so we did not have any data to use to perform a sample size estimation. We therefore based our sample size estimation on work evaluating the change in activity in dogs with OA-pain that were treated with an NSAID. We assumed that the change in activity within dogs with OA-pain may be similar to the difference between healthy dogs and dogs with OA-pain. Our (unpublished) data from 60 dogs with OA-pain that were treated with an NSAID revealed a mean increase in weekly activity of 96,000 (SD 100,000), suggesting 20 dogs per group would be needed to detect this difference with a power of 0.8. We therefore aimed to recruit 20 dogs per group.

Statistical analyses were performed using JMP software (JMP Pro 13; JMP Statistical Discovery, LLC. (Cary, NC) and R (R Foundation for Statistical Computing, Vienna, Austria). All parameters were tested for normality using the goodness fit, and subsequent statistical tests (*t*-Test or Wilcoxon) were used based on normal or non-normal distribution of the data. For the primary objective, *R*^2^ correlation coefficients were calculated to assess for correlation between the two types of devices using paired hourly activity count data for every hour of the 2-week period. Measured parameters were compared between groups using appropriate statistical tests based on distribution of the data. The critical values of the tests were adjusted based on the multiplicity of the comparisons being made and are reported along with results. Due to the reported effects of bodyweight and age on activity in dogs, and differences between the groups in sex distribution, univariate tests of several covariates (OA-pain status, age and body weight, and sex) were performed. Simple linear models were fit with total average activity as the response and each of age, body condition score (BCS), sex, spay/neuter status, and weight (kg) as fixed effects in separate models to select candidate covariates for a model including OA status. Variables were selected for entry if they had a *p*-value < 0.10 in these single-covariate models. A larger model was then fit with those variables and OA status as predictors of total average activity. Backward selection via Akaike information criteria (AIC) was then applied to remove any variables which were not useful in this larger model. As AIC is not useful for model selection in the case of highly related, collinear predictors, we first calculated the variance inflation factors (VIF) for the variables in the larger model and considered for removal any with excessive values (VIF >4). Correlation coefficients (r) were also calculated to explore relationships between the PetPace collar output parameters and CMI questionnaire scores, orthopedic examination scores, and other parameters. Strength of correlation was based on: negligible (0.0–0.3), low (0.3–0.5), moderate (0.50–0.7), high (0.7–0.9) and very high (0.9–1.0) (25).

## Results

### Subjects

**Figure 3:**
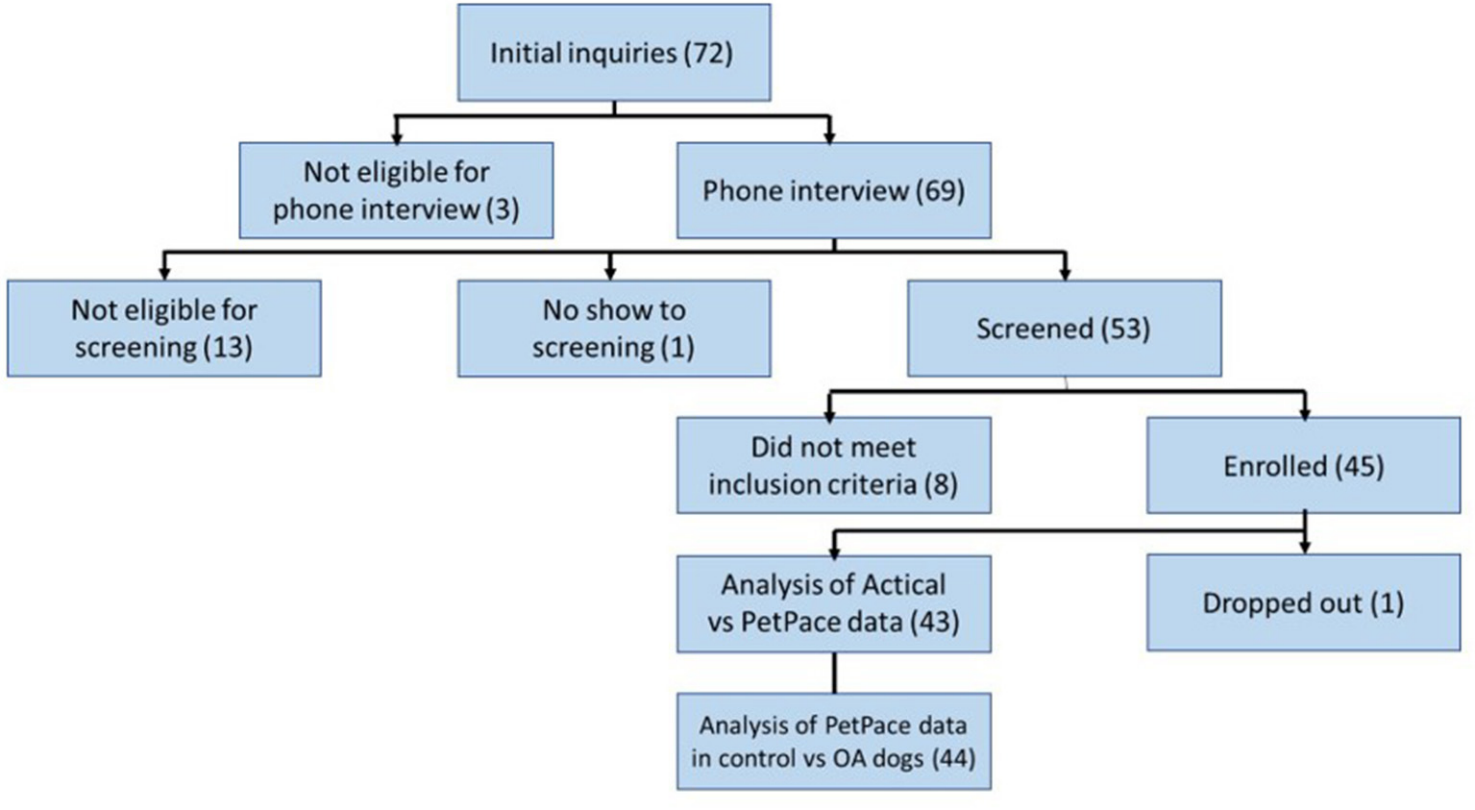
Study participant flow diagram.

Details of the dogs screened and included in the study are shown in Figure 3. Twenty-two healthy dogs and 23 OA-pain dogs were enrolled. One of these 45 dogs was removed from the study on day 8 due to pruritis and discomfort related to the collar. In this dog, the first 7 days of data were retained in the analysis. In one dog, the Actical malfunctioned and only PetPace data were collected. In another dog, PetPace data were not collected due to a collar registration error: while setting up the dog profile on the PetPace website, one subject was mistakenly set up as a cat, and due to the algorithm this triggered, only the activity data could be used. Therefore, data from 43 of 45 enrolled dogs were used to compare Actical and PetPace output, and data from 44 dogs were used to evaluate the discriminatory ability of PetPace to detect differences between dogs with OA pain and healthy dogs.

Demographic characteristics of dogs enrolled in the study are shown in Table 1. There were no differences in body weight (*p* = 0.924) or BCS (*p* = 0.25) between groups. Dogs in the OA group were older than healthy dogs (*p* < 0.0001) despite active efforts to recruit older dogs to the healthy population. Sex distribution in healthy dogs differed from that in dogs with OA pain (*X*^2^ = 0.014). All CMIs detected a difference between the OA and healthy groups (Table 1), indicating that the OA-pain group had pain and functional impairment. Day 14 CMI scores did not differ from Day 0 CMI scores (data not shown).

**Table 1:** Demographic information of dogs in the healthy and OA-pain groups.

**Parameter**		**Healthy (*n* = 22)**	**OA-pain (*n* = 23)**	***P*** **-value**
**Age (yr)**		5.59 ± 2.95	9.78 ± 3.62	**0.0001**
Mean ± SD		(5.5, 1–12)	(10, 4–17)	
(Median, range)
**Weight (kg)**		28.9 ± 8.8	29.2 ± 8.3	0.924
Mean ± SD		(26.9, 11.7–43.8)	(31.4, 12.2–40.4)	
(Median, range)
**BCS**		4.3 ± 0.7	4.7 ± 0.8	0.25
Mean ± SD		(4, 4–7)	(5, 4–6)	
(Median, range)
**Sex**	M	0 (0)	1 (4.3)	
*n* (% total)	MN	10 (45.4)	6 (26.1)	*X*^2^ =
	F	4 (18.2)	0 (0)	**0.014**
	FS	8 (36.4)	16 (69.6)	
**Examinations**	Total pain score	0 ± 0 (0, 0−0)	4.8 ± 2.3 (4, 2−12)	**<0.0001**
**and CMIs**	LOAD	5.0 ± 2.9	22.6 ± 7.9	**<0.0001**
Mean ± SD		(5, 1–11)	(21, 6–39)	
(Median, range)	CBPI pain	0.13 ± 0.30	3.45 ± 2.17	**0.0352**
		(0, 0–1.25)	(3, 0–7.75)	
	CBPI function	0.09 ± 0.30	3.96 ± 2.88	**<0.0001**
		(0, 0–1.17)	(2.8, 0–9.83)	
	SNoRE	2.85 ± 1.11	3.5 ± 1.38	**0.042**
		(2,3, 1.4–5.4)	(2.8, 1–6.8)	

yr, years; SD, standard deviation; kg, kilograms; BCS, body condition score; M, male; MN, male neutered; F, female; FS, female spayed; CMI, Clinical Metrology Instruments; LOAD, Liverpool in Osteoarthritis in Dogs questionnaire; CBPI, Canine Brief Pain Inventory questionnaire; SNoRE, Sleep Nighttime and Restlessness Evaluation Score questionnaire. Bolded *p*-values indicate significance.

### Accelerometry

#### Missing data

Over the 14-day period of the study, the maximum number of hours of data that could be collected was 336 for each dog. The mean time the collar was on each dog was 325 (±7.3) hours. The mean time lost per dog (data not collected) was 12.66 h in healthy dogs (3.9% of mean total) and 10.63 h in OA dogs (3.3% of total). Based on owner diary entries, the main cause of missing data was the need to charge the PetPace battery (2–3 h per charge). Another reason for missing data was that the collar was removed from the dog for bathing or swimming. Once the timespans over which missing data were identified from either device, or both, data were excluded for these times for both devices such that only data collected at the same time from both devices were included. The hours of data collected and used for analysis are summarized in Supplementary Table 1.

#### Comparison of accelerometer output from Actical and PetPace

**Figure 4:**
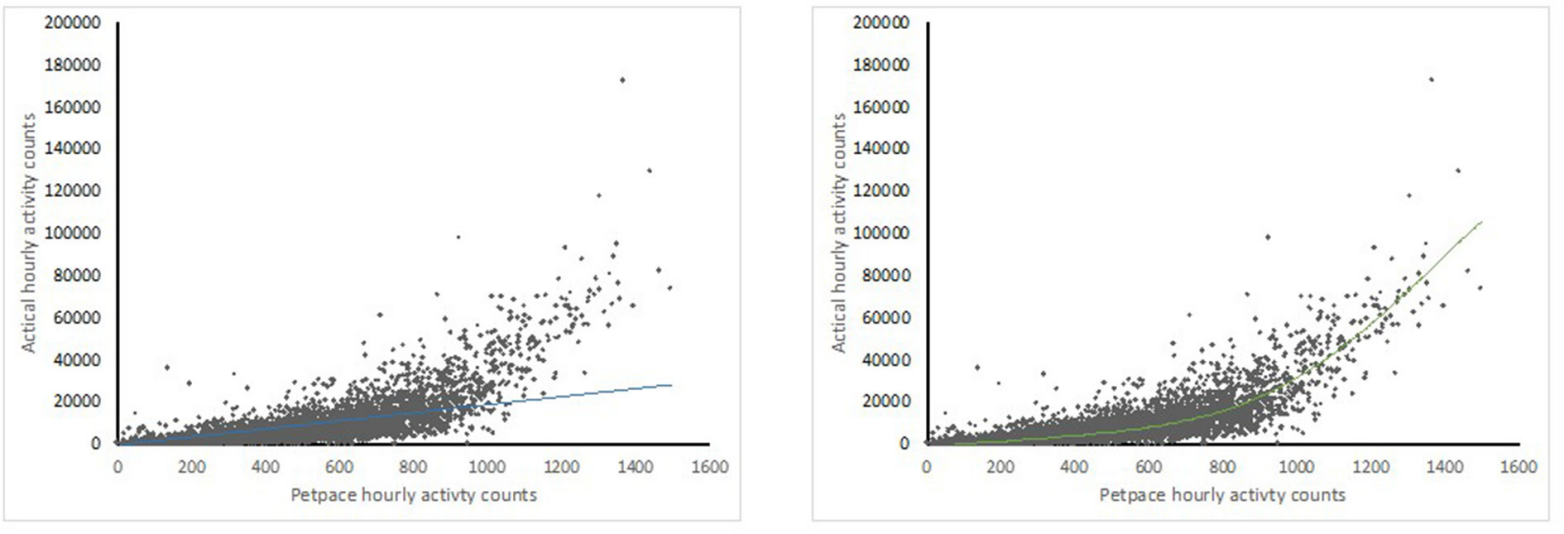
Scatter plot graphs showing correlations between sums of activity counts per hours between Actical (y) and PetPace (x) monitors with linear (left) and 5th order polynomal (right) best fit lines.

Data from 43 dogs (both the healthy and OA-pain groups) were used to explore the relationship between Actical and PetPace output. There was a moderate correlation between the Actical and PetPace monitors (*R*^2^ = 0.56, *p* < 0.001) in the linear model. Correlation improved once a polynomial best fit line was used (*R*^2^ = 0.79, *p* < 0.0001) (Figure 4).

#### Discriminatory ability of the PetPace collar output to detect the effects of OA-pain on dogs' activity

The mean hourly activity counts were statistically significantly higher for the healthy dogs than for the OA-pain dogs, and the mean hourly activity count for the latter group was 22.1% lower over the 14-day study period (Table 2). Looking at candidate covariates, we found age (*p* = 0.012) and spay/neuter status (*p* = 0.004) had a significant effect on activity. Backward selection with AIC removed age from our model. The final model after the elimination of age showed a significant effect of OA-pain status (*p* = 0.004, estimate −54.4) and of spay/neuter status (*p* = 0.016, estimate −70.8, with negative values indicating lower activity in spayed/neutered dogs).

**Table 2:** Hourly PetPace activity counts for each group.

	**Healthy (*n* = 21)**		**OA (*n* = 23)**		***P*** **-value**	**Test**
	**Mean ±SD**	**Median, range**	**Mean ±SD**	**Median, range**		
Average hourly activity count	293 ± 76	299, 187–459	228 ± 43	235, 151–329	0.005	Wilcoxon

SD, standard deviation.

The OA-pain dogs had a higher number of zero activity counts (epochs where no activity was recorded) detected overall (*p* = 0.029 for daytime hours, *p* = 0.063 for nighttime hours), indicating the OA-pain dogs spent more time inactive compared to the healthy dogs (Table 3). There were approximately 10% more zero readings during daytime hours for the OA-pain dogs than for healthy dogs. OA-pain dogs were 22% less active (i.e., more inactive) during the day than the healthy dogs.

**Table 3:** Percentage of PetPace reported epochs that returned zero activity counts during daytime and nighttime.

	**Healthy (*n* = 21)**		**OA (*n* = 23)**		***P*** **-value**	**Test**
	**Mean ±SD**	**Median, range**	**Mean ±SD**	**Median, range**		
%0 counts during daytime	45.2 ± 9.1	46.6, 29.5–60.3	51.6 ± 7.5	52.1, 35.0–66.8	**0.029**	Wilcoxon
%0 counts during nighttime	78.5 ± 6.4	79.1, 56.9–89.2	80.4 ± 8.3	83.5, 50.9–89.1	0.063	Wilcoxon

SD, standard deviation. Bolded *p*-values indicate significance.

With respect to PetPace-defined levels of activity, overall, OA-pain dogs spent more time resting and less time in low, medium, and high activity levels during the daytime, nighttime, weekday, and weekend intervals evaluated (Table 4). Given the multiplicity of activity level and time interval comparisons (16 healthy to OA-pain group comparisons), the critical *p*-value was set at 0.0031. Healthy dogs spent significantly more time in high activity levels than OA-pain dogs across all partitions of the data, and OA-pain dogs spent significantly more time resting than healthy dogs during nighttime. Overall, all dogs spent most of their time resting, with OA-pain dogs spending 3.1% more time resting than the healthy dogs. The numerical differences between the groups for the percentages of time spent in different levels of activity were small, especially for high levels of activity. However, healthy dogs spent 72, 82, 69, and 42% more time in high activity than OA-pain dogs during the daytime, nighttime, weekday, and weekend partitions of the data.

**Table 4:** Percentage of time spent in different activity levels (as defined by Petpace output) during the day, night, weekday, and weekends.

	**Healthy (*n* = 21)**		**OA (*n* = 22)**		***P*** **-value**	**Δ**	**Test**
	**Mean ±SD**	**Median, range**	**Mean ±SD**	**Median, range**			
D rest	84.9 ± 4.8	85.4, 71.2–93.5	88.6 ± 3.0	88.4, 82.3–94.0	0.004		Wilcoxon
D low	3.9 ± 1.1	3.8, 1.8–5.9	3.4 ± 1.0	3.1, 2.0–5.9	0.689		*t*-test
D medium	7.5 ± 2.3	7.1, 3.3–13.5	5.8 ± 1.6	5.9, 3.1–9.3	0.004		*t*-test
D high	3.7 ± 1.7	3.5, 1.5–9.3	2.2 ± 0.8	2.1, 0.8–3.5	**0.0002**	71.9	Wilcoxon
N rest	96.8 ± 1.1	96.9, 94.8–98.5	97.8 ± 0.7	97.8, 96.3–98.8	**0.0009**		Wilcoxon
N low	0.9 ± 0.3	1.0, 0.4–1.6	0.7 ± 0.2	0.6, 0.3–1.1	0.024		*t*-test
N medium	1.6 ± 0.6	1.6, 0.7–2.7	1.1 ± 0.3	1.1, 0.6–1.8	**0.001**		Wilcoxon
N high	0.7 ± 0.2	0.7, 0.4–1.4	0.4 ± 0.2	0.3, 0.1–0.8	**0.0001**	81.6	Wilcoxon
WD rest	89.6 ± 3.5	90.3, 81.2–94.5	92.2 ± 2.0	92.3, 86.7–95.6	0.006		Wilcoxon
WD low	2.7 ± 0.8	2.7, 1.5–4.6	2.3 ± 0.6	2.2, 1.4–3.7	0.077		*t*-test
WD medium	5.2 ± 1.7	4.9, 2.7–9.3	4.0 ± 1.1	4.0, 2.1–6.9	0.01		Wilcoxon
WD high	2.5 ± 1.2	2.4, 1.3–6.4	1.5 ± 0.6	1.5, 0.6–2.7	**0.0004**	69.3	Wilcoxon
WE rest	88.3 ± 4.6	88.4, 75.3–97.6	91.0 ± 2,3	91.1, 86.5–95.1	0.01		Wilcoxon
WE low	3.0 ± 1.1	2.8, 0.6–5.4	2.7 ± 0.8	2.6, 1.6–4.7	0.326		*t*-test
WE medium	5.8 ± 2.3	5.7, 1.3–12.0	4.6 ± 1.2	4.5, 2.5–7.1	0.025		Wilcoxon
WE high	2.9 ± 1.4	3.0, 0.5–7.4	1.7 ± 0.6	1.7, 0.7–2.6	**0.0004**	42.0	Wilcoxon

SD, standard deviation; D, daytime; N, nighttime; WD, weekday; WE, weekend. Bolded *P*-values indicate statistical significance based upon the critical *P*-value (0.0031) used to adjust for the 16 statistical comparisons conducted on this data set. Δ denotes the percent difference in activity counts between groups, calculated from [(mean of healthy—mean of OA-pain)/(mean of OA-pain)] × 100.

##### Position

According to PetPace's proprietary algorithm that determines the body position, several differences were detected between healthy and OA-pain dogs (Table 5). Overall, OA-pain dogs spent more time lying down and less time standing than the healthy dogs. Given the multiplicity of activity level and time interval comparisons (16 healthy to OA-pain group comparisons), the critical *p*-value was set at 0.0031. Thus, the only significant difference was that OA-pain dogs spent less time in a standing position during the day than the healthy dogs. This difference was equivalent to the OA-pain dogs spending 45% less time standing compared to that of the healthy dogs, although the percentage of time each day spent standing was small (3–6%).

**Table 5:** Mean time spent in each position (expressed as a percentage of the total time a position was recorded by PetPace) for daytime and nighttime periods.

	**Healthy (*n* = 21)**		**OA (*n* = 23)**		***P*** **-value**	**Test**
	**Mean ± SD**	**Median, range**	**Mean ± SD**	**Median, range**		
D lying left	5.3 ± 3.2	4.2, 1.0–12.1	6.6 ± 3.4	7.5, 0.2–12.1	0.203	*t*-test
D lying right	7.6 ± 3.8	8.1, 1.2–15.0	9.9 ± 4.8	9.9, 0.6–19.4	0.095	*t*-test
D lying sternal	63.1 ± 8.8	64.0, 47.1–80.6	67.9 ± 9.2	66.8, 54.3–90.4	0.09	*t*-test
D sitting	12.8 ± 6.9	10.8, 3.6–27.8	9.2 ± 6.2	7.2, 0.3–20.0	0.082	Wilcoxon
D eating	2.5 ± 2.6	0.9, 0.1–8.5	1.2 ± 0.9	1.0, 0.1–3.8	0.388	Wilcoxon
D standing	6.4 ± 3.4	5.7, 2.3–15.8	3.5 ± 1.6	3.6, 1.0–8.4	**0.002**	Wilcoxon
D lying on the back	2.2 ± 2.9	0.9, 0.1–10.1	1.7 ± 1.6	1.2, 0–5.8	0.846	Wilcoxon
N lying left	11.4, 5.2	9.9, 3.5–20.7	11.4, 4.6	10.5, 4.2–21.3	1	Wilcoxon
N lying right	14.8 ± 7.6	13.3, 4.9–28.2	15.1 ± 9.4	14.6, 1.2–37.3	0.9	*t*-test
N lying sternal	62.9 ± 15.2	63.1, 19.3–83.8	64.8 ± 13.3	63.4, 39.4–89.6	0.66	*t*-test
N sitting	3.7 ± 3.1	2.2, 0.9–11.6	1.9 ± 1.7	1.4, 0.1–6.5	0.014	Wilcoxon
N eating	0.8 ± 0.9	0.6, 0.0–3.5	0.9 ± 1.6	0.2, 0.0–6.8	0.395	Wilcoxon
N standing	1.3 ± 2.1	0.7, 0.1–10.0	0.5 ± 0.6	0.4, 0.1–2.9	0.0084	Wilcoxon
N lying on the back	5.1 ± 7.8	2.1, 0.0–30.0	5.4 ± 7.6	2.7, 0.0–35.2	0.761	Wilcoxon
D SUM lying	78.3 ± 10.2	79.8, 56.6–92.1	86.1 ± 6.6	87.7, 75.1–97.1	0.006	*t*-test
N SUM lying	94.2 ± 5.2	95.7, 74.9–98.5	96.7 ± 2,2	97.1, 91.8–99.7	0.051	Wilcoxon

SD, standard deviation; D, daytime; N, nighttime; SUM, summary. The bolded *P*-value indicates statistical significance based the critical *P*-value (0.0031) used to adjust for the 16 statistical comparisons conducted on this data set.

##### Nighttime activity

The average activity counts per hour at nighttime in the healthy dogs were higher than in the OA-pain dogs (106.09 ± 28.02 vs. 77.55 ± 19.64, respectively; *p* = 0.004).

##### Heart rate variability parameters

All HRV parameters in OA-pain dogs were numerically lower than in healthy dogs (Table 6). Across the calculated HRV parameters (*n* = 5), there were 10 comparisons between the groups so the adjusted critical *p*-value is 0.01, meaning that only VVTI, SDANN, and SDANN Index in OA-pain dogs were significantly lower than in healthy dogs.

**Table 6:** Heart rate variability parameter measurements.

	**Healthy (*n* = 21)**		**OA (*n* = 22)**		***P*** **-value**	**Test**
	**Mean ± SD**	**Median, range**	**Mean ± SD**	**Median, range**		
VVTI	11.6 ± 0.2	11.6, 11.2–11.8	11.4 ± 0.1	11.4, 11.1–11.6	**0.0018**	*t*-test
SDANN (ms)	126.2 ± 54.0	127.5, 22.6–210.4	96.7 ± 48.1	89.5, 30.8–199.4	0.077	*t*-test
SDANN index (ms)	325.6 ± 23.5	331.0, 280.5–363.1	303.1 ± 17.7	299.7, 258.0–329.4	**0.001**	*t*-test
SDNN (ms)	353.5 ± 36.2	355.6, 292.1–421.6	323.0 ± 30.2	318.3, 265.8–381.6	**0.0048**	*t*-test
Triangular index	83.3 ± 19.3	83.3, 54.6–112.6	69.0 ± 18.4	66.9, 39.0–109.4	0.026	Wilcoxon

Bolded *P*-values indicate statistical significance based upon the critical *P*-value (0.01) used to adjust for the 10 statistical comparisons conducted on this data set. ms, milliseconds.

##### Pulse and respiratory rates

Recorded pulse rate values did not differ between groups (Table 7). Recorded respiratory rates did not differ betweeen the groups (data not shown).

**Table 7:** Pulse measured by the PetPace device.

		**Healthy**		**OA**		**Test**	***P*** -value^*^
		**Mean ± SD**	**Median, range**	**Mean ± SD**	**Median, range**		
Daytime	Pulse Min	44 ± 9		49 ± 8		Wilcoxon	0.033
	Pulse Max	117 ± 30		119 ± 26		*t*-test	0.812
	Pulse Av	66 ± 5		70 ± 6		*t*-test	0.013
Nighttime	Pulse Min	43 ± 11		52 ± 11		Wilcoxon	0.013
	Pulse Max	82 ± 16		86 ± 24		Wilcoxon	0.326
	Pulse Av	62 ± 7		64 ± 6		Wilcoxon	0.197

Min, minimum; Max, maximum; Av, average; SD, standard deviation.

* None of these values were below the critical *P*-value (0.01) used to adjust for the 10 statistical comparisons conducted on this data set.

##### Correlation between PetPace-measured activity and subjective assessments

There was poor to low (but significant) negative correlation between CMI scores at Day 0 and the average hourly activity counts from the PetPace collar (Table 8), i.e., dogs with higher CMI scores tended to have lower activity counts. The largest negative correlation between the average hourly activity counts and the CMI scores was found for the LOAD score. SNoRE values did not appear to correlate with average hourly activity counts or average hourly nighttime activity counts.

**Table 8:** Correlation between activity measured by the PetPace monitor (mean hourly activity counts), CMI scores, and total joint pain scores on Day 0.

**Comparison**	***r***	***P*** **-value**
LOAD	−0.33	**0.0001**
CBPI pain	−0.23	**0.0001**
CBPI function	−0.25	**0.0001**
Total joint pain	−0.16	**0.0001**
SNoRE	−0.035	0.23
SNoRE^*^	−0.054	0.13

LOAD, Liverpool in Osteoarthritis in Dogs questionnaire; CBPI, Canine Brief Pain Inventory questionnaire; SNoRE, Sleep Nighttime and Restlessness Evaluation Score questionnaire;

* indicates that the comparison was between mean hourly activity counts over the nighttime.

Bolded *p*-values indicate significance.

## Discussion

This study found moderate correlation between the PetPace collar and the Actical monitor output, confirming previous findings (20). Significant differences in both activity counts, time spent in different levels of activity and recorded positions, as measured by the PetPace collar, were noted between healthy dogs and OA-pain dogs. OA-pain dogs had lower overall activity counts than healthy dogs, spent significantly less time at higher activity, and spent significantly less time standing. This is the first study to directly compare the activity of healthy dogs and dogs with untreated OA-pain and is the first study to report position data from the PetPace collar.

There was moderate linear correlation between the average hour activity count outputs from the Actical monitor and the PetPace collar but higher correlation using a polynomial fit, suggesting that the PetPace collar underestimates higher activity levels compared to the Actical monitor. The reason for the relatively lower output from the PetPace collar compared to the Actical monitor noted at higher activity levels is likely due to both the lower sampling rate of the PetPace collar as well as its longer epoch. The PetPace collar samples dogs' activity at 1Hz compared to the Actical monitor's 32Hz, and the PetPace collar's data is the average of the dogs' activity within a 2–3 min period of time compared to the 1 min epoch of the Actical monitor. Therefore, high intensity activities that last <2 min tend to receive a lower integer from the PetPace collar compared to Actical monitor since they are being averaged with other activity during the epoch. In humans, lower sampling rates resulted in lower reported activity counts compared to higher sampling rates (26), and longer epoch lengths resulted in more missed minutes of moderate and high activity than shorter epoch lengths (27). In previous work, activity between the Whistle activity monitor and the Actical monitor were compared, and high correlation was reported (0.81) between summed 3 min outputs from the Actical monitor and the 3 min epoch output provided by the Whistle activity monitor (28). Although not specified in the report, it is assumed that this was a linear correlation (28). In a similar comparative study, the Heyrex activity monitor was compared to the Actical monitor with both the epochs set at 1 min, and the Pearson correlation was found to be 0.87 (29). In this latter study, the relationship between output appeared linear [see Figure 1 of (29)].

Previous studies have evaluated differences in activity between healthy cats and cats with OA-pain (15). Previous work of ours reported that dogs assessed by owners to be more impaired from OA had overall lower total weekly activity counts as measured using Actical monitors (30). This current report is the first study to directly compare activity levels between healthy dogs and dogs with OA-pain. Using the PetPace collar activity count output, we found that OA-pain dogs had lower activity than healthy dogs with less time being spent in all levels of activity (rest, low, medium, and high, as determined by PetPace's proprietary algorithms) across daytime, nighttime, weekends, and weekdays. The lower activity in OA-pain dogs seems to have been driven by significantly less time being spent in high intensity activity. This finding has been found in other species as well. A study evaluating human patients with early knee osteoarthritis found that they accumulated little time in vigorous activity (31). Additionally, across all time periods evaluated, OA-pain dogs had more zero counts recorded, suggesting they spent more time inactive than healthy dogs. Humans with OA demonstrate similar findings (32). The decreased activity in OA-pain dogs we documented could be due to a number of factors, none of which are mutually exclusive. OA-pain may be one factor resulting in decreased activity in OA-pain dogs. Studies in humans have shown differences in activity levels between people with and without OA-pain as well as differences in activity dependent on severity of OA. One such study showed that adults with ankle OA walked over 50% fewer steps per day than healthy controls (a significant difference), and patients with more severe disease or bilateral ankle OA walked significantly fewer steps than those with unilateral or less severe disease (33). Some work has evaluated the effect of treatment of OA-pain on activity levels in humans, and found effective treatment increases activity. Frimpong et al. showed that the proportion of time that patients spent in sedentary behavior decreased and the time they spent in light intensity physical activity increased from baseline to 6 months postoperatively following total knee arthroplasty (34). Such data suggest that OA-pain negatively impacts activity levels. Similar conclusions have been reached in dogs and cats in studies where analgesics have been shown to increase activity metrics in analgesic-treated animals compared to placebo-treated animals (13, 15, 17, 19, 35, 36). The aforementioned findings support our current study data suggesting decreased activity in OA-pain dogs compared to healthy dogs: these previous studies have shown that dogs with OA-pain display increased activity when given an analgesic, implying that their “OA-pain state” results in decreased activity.

Other factors may also have influenced the results. Owners may consciously or unconsciously limit their dog's activity if they suspect or know their dog suffers from OA-pain which could account for the differences between the groups. Current information clearly suggests that owner activity likely influences dog activity (37), but to our knowledge no work has evaluated whether owners influence activity of their dog if they know it has OA.

Bodyweight has been shown to affect activity counts in dogs (38). Brown et al. found that total stair-walking activity counts were influenced by body weight. More specifically, there was a 1.7% decrease in activity for every 1 kg increase in a dog's weight (38). The weight range in that study was 25 ± 13 kg, similar, though a bit broader, than the weight range in the current study. In a study of post-surgical activity, smaller dogs had larger decreases in average activity counts after undergoing laparoscopic-assisted gastropexy (39). In our study, there was no difference in weight between groups, suggesting that bodyweight did not affect the results.

No relationship between sex and activity has been established in dogs (38) but it has been well-established in humans that men are consistently more active than women (40) and that men spend more time at higher intensity activity levels than women (41), both of which could be influenced not only by biologic factors but also sociologic and gender role factors that obviously are not present for dogs. In this study, there were more male dogs in the healthy group than in the OA-pain group which could partially account for the lower activity counts found in OA-pain dogs. We did find a significant effect of spay/neuter status, with spayed/neutered dogs having lower activity than intact dogs. There were more spayed/neutered dogs in the OA-pain group, which could have influenced the results. However, in statistical model the effect of OA-pain was still significant, despite the effect of spay/neuter status. Further research with larger sample sizes is warranted to determine if spay/neuter status or sex influences activity levels in dogs with and without OA-pain.

Age has been shown to decrease activity counts in dogs. Older dogs have been shown to have lower activity counts than younger dogs (38). In fact, for every 1 year increase in the dog's age, there was a 4.2% decrease in stair-walking activity (38). Older OA-pain dogs have been shown to have a greater response to NSAID therapy than younger dogs (13). The authors of that study postulated that older dogs may have been more severely affected by their OA-pain and thus had a greater response to treatment. The healthy dogs in this study were significantly younger than the OA-pain dogs despite active recruitment for older, healthy dogs and younger, arthritic dogs. Although OA-pain occurs in young dogs, it is more easily detected by owners in older dogs. In fact, over 50% of osteoarthritis cases are diagnosed in dogs over 8 years of age (42). Due to the higher prevalence of OA detected in older dogs, it can be very difficult to age-match subjects, a struggle that has also been described in human OA studies (33). However, in our analysis, when controlling for age, we still found a significant effect of OA-pain in this study. Further research is needed to fully understand the effects of various factors on activity in both healthy dogs and OA-pain dogs.

The PetPace monitor detected differences between OA-pain dogs and healthy dogs in the amount of time spent in various positions. We found a significant difference in the amount of time spent standing: OA dogs spent less time standing than healthy dogs. To our knowledge there are no published studies validating the output of the PetPace monitor as a measure of position, and concurrent monitor and visually assessed position data were not captured in this study. If the PetPace monitor is a valid tool for assessing position, then, as with the argument above, there may have been an effect of pain, age, and/or weight on the time spent standing.

OA-pain dogs had significantly lower activity counts at night compared to healthy dogs. We had assumed that OA-pain would disrupt sleep and that this would manifest as increased activity, or “restlessness.” There was no evidence of restlessness or increased nighttime activity in OA-pain dogs in this study. In previous studies, NSAID administration to dogs with OA-pain had reduced activity at nighttime, suggesting that OA-pain was driving increased activity over the nighttime period (11, 23). In humans, the connection between OA-pain and sleep disturbances such as insomnia, abnormal sleep EEG pattens, abnormal circadian rhythms, restless leg syndrome, and hypersomnia is well-established (43, 44).

Research into HRV in veterinary medicine is limited; the majority of studies focused on stress states and cardiovascular diseases (45, 46). The HRV parameters derived from data captured by the PetPace collar in this study are the first comparing dogs with and without OA-pain. The HRV parameters were all numerically lower in OA-pain dogs, but the magnitudes of these differences were, for the most part, small. The values obtained in the current study were consistent with pilot data obtained in a small number of dogs with arthritis pain (*n* = 6) and control dogs (*n* = 7) (47). The magnitudes of the significant differences seen in the VVTI and SDANN Index were small, and thus the clinical relevance of these findings is unknown. However, HRV has been shown to be depressed in humans with chronic pain conditions such as arthritis, fibromyalgia, and abdominal pain (48–51) so HRV may become a useful indicator of chronic pain in dogs. Studies in humans have shown that HRV is affected by age, and some evidence exists for this in dogs (52), so the differences between the groups could have been influenced by age. The HRV results in this study warrant verification, and further investigation into the influence of age and other factors on HRV in relation to the influence of OA pain. Determining how HRV changes in response to treatment of chronic pain may provide another useful tool for assess response to therapy.

CMIs have been used in veterinary medicine to differentiate between healthy dogs and those with OA-pain as well as to determine response to treatment for OA-pain. They primarily rely upon owner-perceptions of the dogs' pain, quality of life, and abilities to perform activities but do not directly ask about the dogs' activity levels (9–11). Thus, a non-perfect relationship between CMIs and activity counts, as found in the current study, is not unexpected. The CMI results for the OA-pain dogs in this study are on par with those seen in dogs with untreated OA-pain in similar studies (19, 53, 54). In this study, there was low to no correlation of all CMIs with average total hourly PetPace activity counts. Overall, the LOAD correlated best with the average total hourly PetPace activity counts (*r* = −0.33). Negative correlation is expected because higher LOAD scores indicate a greater degree of mobility impairment which likely decreases activity counts. The CBPI pain interference score (PIS) showed the second-best correlation with the average total hourly PetPace activity counts (*r* = −0.25). That the LOAD and CBPI PIS had the largest correlation is not surprising as they capture information related to activities the dogs perform.

There are several weaknesses of the current study. The first concerns the validity of the PetPace output. Our previous work (20) and the current data showing a moderate correlation between the validated Actical and PetPace outputted activity counts suggests the PetPace accelerometer output is a valid measure of dog movement. However, with respect to other output (physiological parameters and algorithm-derived indices), there are no data publicly available on the accuracy or validity of these outputs. This study took the manufacturer's claims at face value, and future work should be done to validate the output. Regardless, differences were detected between healthy dogs and dogs with OA-pain, compelling further exploration of this. It is unknown whether co-mounting the accelerometers (Actical monitor mounted onto the PetPace collar) impacted the PetPace collar's function. The weight distribution of dogs was narrow, the healthy dogs were younger than the OA-pain dogs, and the sex distribution was not balanced. All these factors should be explored in more detail in subsequent work. An important fact to note is that much of the data used was derived from proprietary algorithms applied to the data by the manufacturers of the PetPace collar, limiting the full understanding of the detected differences between the groups. Additionally, although differences were detected between the groups and attributed to OA-pain, further work using an effective analgesic is needed to determine if the differences are truly due to pain or some other characteristic of the OA-pain cohort.

As this is the first study to compare activity levels between healthy dogs and untreated dogs with OA-pain, additional research is warranted to verify our findings, ideally in more closely matched groups of dogs with respect to age, body condition score, and sex (case-controlled study). Comparing patterns of activity in healthy and OA-pain dogs to see how and when they differ as well as understand how varying degrees of OA-pain impact activity should be pursued as this information, collectively, may point to aspects of activity or activity profiles that could be assessed as potential objective outcome measures in analgesic studies. The use of wearable or implantable sensors could provide veterinarians an objective measure of the effects of pain which could be utilized to measure the effectiveness of various treatments in our efforts to better treat chronic pain in dogs.

## Data availability statement

The raw data supporting the conclusions of this article will be made available by the authors, without undue reservation.

## Ethics statement

The animal study was reviewed and approved by Animal Care and Use Committee at North Carolina State University (IACUC #17-110-O). Written informed consent was obtained from the owners for the participation of their animals in this study.

## Author contributions

BDXL designed the study. BB, JH, and BDXL collected data. BB, BDXL, JR, and ARO contributed to statistical analysis. ARO, BB, JH, ME, JR, and BDXL participated in drafting and revising the manuscript. All authors contributed to the article and approved the submitted version.

## Conflict of interest

Author BDXL has been a paid consultant for Boehringer Ingelheim Animal Health. This work was funded by Boehringer Ingelheim Animal Health (Duluth, GA, USA) (grant number 2017-0917) and the PetPace collars and data were supplied by PetPace Ltd (Burlington, MA, USA). The funders had no involvement in the study design, collection, analysis, interpretation of data, the writing of the article or the decision to submit it for publication. However, the funders were shown the results as a courtesy, and their input/comments were taken into consideration. The remaining authors declare that the research was conducted in the absence of any commercial or financial relationships that could be construed as a potential conflict of interest.

## Publisher's note

All claims expressed in this article are solely those of the authors and do not necessarily represent those of their affiliated organizations, or those of the publisher, the editors and the reviewers. Any product that may be evaluated in this article, or claim that may be made by its manufacturer, is not guaranteed or endorsed by the publisher.
