# Supplementary material for: Initial exploration of the discriminatory ability of the PetPace collar to detect differences in activity and physiological variables between healthy and osteoarthritic dogs
Source: Front Pain Res (Lausanne). 2022 Sep 6;3:949877. doi: 10.3389/fpain.2022.949877 (PMC9485802; doi:10.3389/fpain.2022.949877)
Supplement: Supplementary file 1 [file Data_Sheet_1.docx]

**NC State Translational Research in Pain (TRiP) Joint Evaluation Scoring SystEm (Canine)**

***JESSE(Canine)***

| R Forelimb | **Pain (0-4)** | | **Crepitus (0-2)** | **Effusion (0-2)** | **Thickening (0-2)** | **ROM (0-2)** |
| --- | --- | --- | --- | --- | --- | --- |
| Manus |  | |  |  |  |  |
| Carpus |  | |  |  |  |  |
| Elbow |  | |  |  |  |  |
| Shoulder |  | |  |  |  |  |
| R Hindlimb | **Pain (0-4)** | | **Crepitus (0-2)** | **Effusion (0-2)** | **Thickening (0-2)** | **ROM (0-2)** |
| Pes |  | |  |  |  |  |
| Hock |  | |  |  |  |  |
| Stifle |  | |  |  |  |  |
| Hip |  | |  |  |  |  |
| L Forelimb | **Pain (0-4)** | | **Crepitus (0-2)** | **Effusion (0-2)** | **Thickening (0-2)** | **ROM (0-2)** |
| Manus |  | |  |  |  |  |
| Carpus |  | |  |  |  |  |
| Elbow |  | |  |  |  |  |
| Shoulder |  | |  |  |  |  |
| L Hindlimb | **Pain (0-4)** | | **Crepitus (0-2)** | **Effusion (0-2)** | **Thickening (0-2)** | **ROM (0-2)** |
| Pes |  | |  |  |  |  |
| Hock |  | |  |  |  |  |
| Stifle |  | |  |  |  |  |
| Hip |  | |  |  |  |  |
| Spinal Column | | **Pain (0-4)** |  |  |  |  |
| Cervical | |  |  |  |  |  |
| Thoracic | |  |  |  |  |  |
| T-L | |  |  |  |  |  |
| Lumbar | |  |  |  |  |  |
| L-S | |  |  |  |  |  |

The descriptors on the following scoring systems are considered a guide and are especially useful for studies and situations where different individuals are making assessments on the same patient over time. Ideally, a single trained individual makes all the assessments on every patient in a given study.

**Pain scale based on passive flexion, extension and manipulation**

0: Does not notice manipulation

1: Orients to site on manipulation, does not resist or only mild resistance (*mild*)

2: Orients to site, slight objection to manipulation (*moderate*)

3: Withdraws from manipulation, may vocalize, may turn to guard area (*significant*)

4: Tried to escape from manipulation, or prevent manipulation, may bite or show aggression on

manipulation (*severe*)

**Crepitus, Effusion, Thickening and Range of Motion (ROM) based on passive flexion, extension and manipulation**:

**Crepitus:**

0: none

1: mild, occasional crepitus

2: moderate, crepitus felt always

3: severe, can feel and hear crepitus

**Effusion:**

0: none

1: mild, small fluid pocket felt only on careful palpation

2: moderate, prominent/obvious on palpation

3: severe, may see visible fluid pocket

**Thickening:**

0: none, can feel all anatomic structures easily

1: mild, less defined anatomic structures

2: moderate, can still discern the detail of anatomic structures of the joint

3: severe, can no longer feel anatomic structures of the joint

**Range of motion:**

0: normal

1: mild-moderate decrease

2: severely decreased
