# Supplementary material for: Initial exploration of the discriminatory ability of the PetPace collar to detect differences in activity and physiological variables between healthy and osteoarthritic dogs
Source: Front Pain Res (Lausanne). 2022 Sep 6;3:949877. doi: 10.3389/fpain.2022.949877 (PMC9485802; doi:10.3389/fpain.2022.949877)
Supplement: Supplementary file 2 [file Table_1.docx]

|  | Control (n = 22) | | | | OA (n = 23) | | | | Test | P-value |
| --- | --- | --- | --- | --- | --- | --- | --- | --- | --- | --- |
|  | **Mean**± **SD** | **Median** | **Min** | **Max** | **Mean**± **SD** | **Median** | **Min** | **Max** |  |  |
| Total hours | 325±8 | 325 | 301 | 355 | 324±7 | 324 | 297 | 336 | Wilcoxon | 0.95 |
| Hours lost | 13±14 | 9 | 2 | 69 | 11±7 | 9 | 2 | 29 | Wilcoxon | 0.76 |
| Study hours | 313±9 | 314 | 286 | 326 | 313±12 | 315 | 268 | 325 | Wilcoxon | 0.49 |
| Night hours | 115±19 | 114 | 90 | 151 | 109±17 | 106 | 86 | 139 | Wilcoxon | 0.97 |
| Daytime hours | 198±18 | 197 | 163 | 230 | 204±16 | 204 | 173 | 228 | T-test | 0.28 |
| Weekdays hours | 222±7 | 223 | 203 | 240 | 220±11 | 224 | 179 | 229 | Wilcoxon | 0.76 |
| Weekends hours | 91±10 | 94 | 54 | 96 | 93±3 | 95 | 87 | 96 | Wilcoxon | 0.88 |

Supplementary Table 1: Summary of mean and SD, minimum and maximum of study hours between groups
